# Fifty years of BMT: risk stratification, donor matching, and stem cell collection for transplantation

**DOI:** 10.3389/fonc.2023.1196564

**Published:** 2023-08-28

**Authors:** Amandeep Salhotra, Shan Yuan, Haris Ali

**Affiliations:** ^1^ Department of Hematology and Hematopoietic Cell Transplantation (HCT), City of Hope National Medical Center, Duarte, CA, United States; ^2^ Division of Transfusion Medicine, Department of Pathology and Laboratory Medicine, City of Hope National Medical Center, Duarte, CA, United States

**Keywords:** disease risk assessment, donor selection criteria, stem cell collection, comorbidity [MeSH], allogeneic stem cell transplantation

## Abstract

In this review, we discuss recipient risk assessment for allo-HCT regarding comorbidities present at baseline to predict non relapse mortality. We further reviewed the incorporation of remission status and cytogenetic risk prior to allograft transplantation to predict relapse rates for hematologic malignancies. HCT-CI and DRI are tools available to physicians to assess the risk–benefit of allo-HCT in patients referred for transplantation. Next, we discuss our algorithm for donor selection and criteria for donor selection in case matched donors are not available. Finally, we discuss our approach for stem cell mobilization, especially in donors failing G-CSF, and our approach for the use of plerixafor and data supporting its use.

## Introduction

Approximately 80,000 allogenic hematopoietic stem cell transplantations (allo-HCT) are performed annually worldwide (1). Allo-HCT remains the most effective treatment for the management of advanced hematologic malignancies and is increasingly used in the management of non-malignant conditions (2). Demographic trends suggest that allo-HCTs are increasingly being performed in patients who are older, have more comorbidities, and have increased utilization of matched unrelated donors based on data published by the Centers for International Blood and Marrow Transplant Research (CIBMTR). With increasing patient age, disease-specific features make primary malignancies more resistant to conventional therapies (3). This combined with an increase in comorbidities in older patients results in an increase in relapse and non-relapse mortality (NRM). Accurate assessment of disease- and patient-specific risk factors that may impact long-term survival is critical and needs to be discussed with patients and their family members at the time of transplant consultation. In this review, we discuss clinically validated tools commonly used for risk stratification in patients undergoing allogenic stem cell transplantation, specifically focusing on the hematopoietic cell transplantation-comorbidity index (HCT-CI) and the disease risk index (DRI), which are the most commonly used to assess NRM and relapse mortality. One of the critical features for the success of allo-HCT is donor selection using multiple options available (matched donor, haploidentical, mismatched, and cord). We discuss our approach towards donor selection for HCT recipients, and in the final section, we discuss our approach for CD34^+^ stem cell collection from healthy donors in allo-HCT and auto-HCT settings, and our approach to HPC collection in difficult cases.

## Demographic trends in allo-HCT

**Figure 1:**
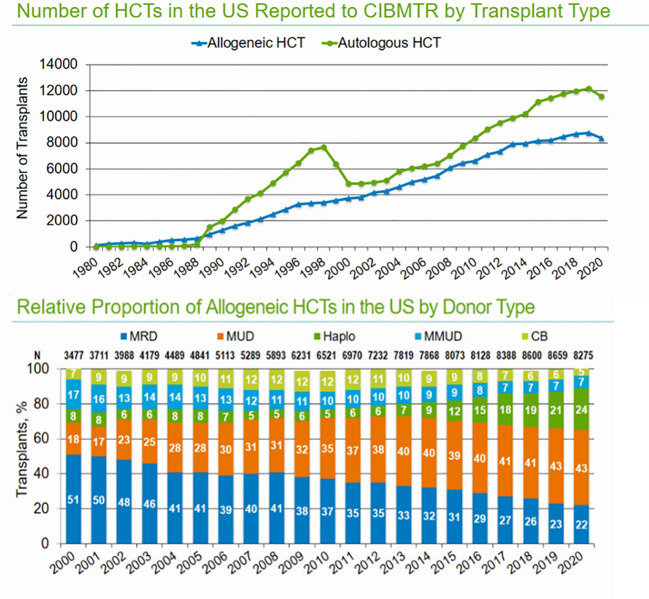
Number of allo-HCTs in the US annually and distribution based on donor types (source CIBMTR summary slides 2021).

**Figure 2:**
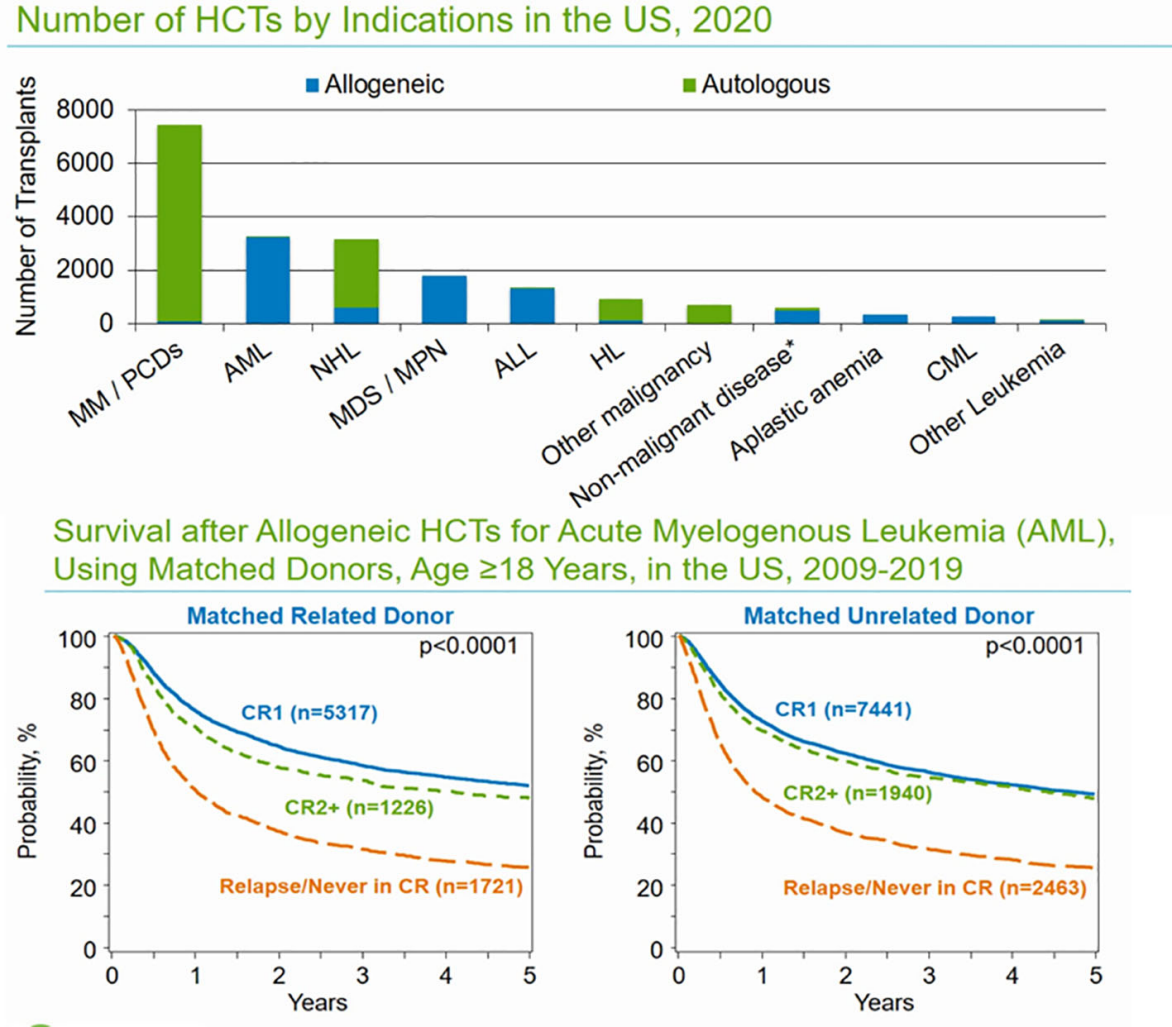
Common indications for auto- and allo-HCTs in the US and overall survival outcomes based on remission status in MRD and MUD.

The Center for International Blood and Marrow Transplant Research (CIBMTR) is responsible for data collection on US and international patients undergoing allogeneic hematopoietic cell transplantation (Allo-HCT) as part of the Stem Cell Therapeutic and Research Act, which was first established in 2005. The CIBMTR database remains the most reliable source for evaluating the trends and outcomes in allo-HCTs. Based on the most recent data available for the reporting period ending in 2021, 8,000 allogenic stem cell transplants were performed in the United States. The majority of allo-HCTs (43%) were performed using a matched unrelated donor (MUD), which now exceeds the number of matched related-donor transplants (22%). Demographic trends show increasing utilization of haploidentical donors (24%), which were used in approximately 2,000 allo-HCTs, slightly more than the total number of matched sibling donor (MSD) transplants (22%) performed in the last reporting period of 2021 (**Figure 1**). With the increasing use of haploidentical donors, the utilization of cord blood (CB) transplants and mismatched unrelated donor transplants (MMUD) continues to decline, and fewer than 500 such transplants were performed in the reporting period in the US (<10%). In both MUD and MRD, peripheral blood stem cell (PBSC) grafts are used most often in adults; however, bone marrow grafts continue to be used in the pediatric population. Calcineurin inhibitor (CNI)-based GVHD prophylaxis is the most used prophylactic regimen in MUD and MRDs, and post-transplant cyclophosphamide (PTCy) is most used in haploidentical HCTs. The use of PTCy is increasing in MUD HCTs, and 26% of MUD-allo-HCTs used this form of GVHD prophylaxis. Use of PTCy is increasing in MUD HCTs and 26% MUD-allo-HCTs used this form of GVHD prophylaxis. For MMUD, PTCy-based GVHD prophylaxis was used in 55% of patients, based on studies showing good control of GVHD. For the most recent reporting period of 2021, the age distribution of patients undergoing allo-HCT was as follows: ≤18 years (9%), 18–39 years (17%), 40–64 years (46%), and ≥65 years (27%). The largest growth in transplant recipients in recent years has been observed in patients aged >65 years. The most common indication for allo-HCT is acute myeloid leukemia (AML), followed by myelodysplastic syndrome/myeloproliferative neoplasm (MDS/MPN) and acute lymphoblastic leukemia (ALL) (**Figure 2**). Less common indications include aplastic anemia (AA), chronic myeloid leukemia (CML), and non-malignant diseases. For AML/MDS patients undergoing myeloablative conditioning (MAC) regimens based on allo-HCT, busulfan and fludarabine (43%) remain the most popular regimens, followed by busulfan cyclophosphamide (28%). Fractionated total-body irradiation (FTBI)-based regimens are used in 21% of MAC HCT recipients. Fludarabine and melphalan are the most frequently used reduced-intensity conditioning (RIC) regimens in 35% of patients, followed by busulfan and fludarabine (27%). Among adult patients with AML receiving MRD transplantation between 2009 and 2019, the 3-year probabilities (95% CI) of survival following transplantation with a disease status of CR1, CR2+ (2nd or subsequent complete remission), and relapsed disease/never in CR (includes primary induction failure) were 58% (57%–60%), 54% (51%–57%), and 31% (29%–33%), respectively.

Among adult patients with AML receiving MUD transplants between 2009 and 2019, the 3-year probabilities (95% CI) of survival following transplantation with a disease status of CR1, CR2+ (2nd or subsequent complete remission), and telapsed disease/never in CR (including primary induction failure) were 56% (55%–57%), 54% (52%–57%), and 31% (30%–33%), respectively.

Approximately 800 allo-HCTs are performed annually in the City of Hope, and AML is the leading indication for allo-HCT. Our standard MAC regimen used was FTBI with cyclophosphamide or etoposide. Using this regimen, the 6-year OS rate was 60% and the non-relapse mortality rate was 15%. Chronic GVHD rates were relatively high at 70%, resulting in a 1-year GRFS rate of 45%. For older patients not eligible for intensive conditioning, Flu/Mel-based RIC is the preferred regimen. Our standard GVHD prophylaxis uses tacrolimus and sirolimus (T/S), based on promising studies performed initially at the Dana Farber Cancer Institute 96 and further phase 2 studies at COH. In the City of Hope, we were the first to use the Flu/Mel regimen in combination with T/S prophylaxis initially in the MRD setting. The aGVHD rates (gd 2–4) were 29% and NRM at day + 100 was 6%, confirming the efficacy and safety of T/S prophylaxis with Flu/Mel in a matched related donor (MRD) setting (4). Subsequently, promising results with T/S prophylaxis were observed when used in combination with MAC regimens in the MRD setting (5). We also observed less mucositis and IPS with T/S use, but an increase in TMA as reported initially by DFCI-but only in combination with Bu/Cy regimen (55%), and TMA with Flu/Mel was only 7%. Based on these promising results, T/S has been the standard of care for GVHD prophylaxis in patients undergoing allo-HCT with either RIC/MAC regimen in matched donors since 2005 (6, 7). Subsequently the safety and efficacy of T/S-based GVHD prophylaxis were demonstrated in both MAC and RIC settings in additional centers (8, 9).

In patients with active leukemia, we developed a novel intensified conditioning regimen comprising total marrow and lymphoid irradiation (TMLI) used in combination with systemic chemotherapy and matched donors. Using this regimen, we observed 1-year overall survival rates greater than 55% in high-risk populations (10, 11). Further advancements for the prevention of GVHD include graft manipulation strategies (12), use of PTCy in mismatched donors (13), and JAK inhibitors in combination with T/S in matched donor settings, resulting in superior 1 year GVHD and relapse-free survival (GRFS) outcomes (14).

## Assessment of comorbidities in patients undergoing allogenic stem cell transplantation

### Comorbidity indices

Comorbidities are any distinct or additional clinical condition that may occur during the clinical course of a patient with a primary disease (15). The existence of comorbid conditions may impact the prognosis, intensity and type of chemotherapy offered, thus having implications for the clinical outcomes of the underlying malignancy. The majority of older patients being considered for treatment have pre-existing conditions that impact clinical outcomes (16). The Charlson Comorbidity Index (CCI) was used to study the interactions between comorbidities and the primary disease. The CCI assigned weighted scores to 19 chronic medical conditions and studied their impact on 1-year mortality in 559 patients admitted to a general Medical Center (17). The CCI was subsequently validated in patients with other medical conditions and solid tumors, and it was correctly able to predict 1-year mortality in multiple clinical scenarios (18–22). Further refinements included adding age ≥50 years as another risk factor to the model (23). Subsequently, CCI was used to successfully predict 1-year mortality in patients undergoing allo-HCT (22). Despite the utility of this tool in predicting mortality of allo-HCT, there were some shortcomings in the CCI, as the tool was primarily developed for patients admitted to the general medical ward. Hence, some of the comorbid conditions used for the calculation of CCI (e.g., advanced heart failure or severe pulmonary compromise) could not be used for allo-HCT patients, because these health conditions would preclude HCT in patients with advanced illness. Additionally, CCI lacks some comorbidities specific to patients undergoing HCT, such as prior infections and psychiatric disturbances that have a bearing on HCT outcomes. Considering these shortcomings and to further improve the accuracy of prediction tools, investigators at the Fred Hutch Cancer Research Center developed the hematopoietic cell transplantation comorbidity index (HCT-CI) (24). For the development of this new tool, 1,055 patients who underwent allo-HCT using either reduced-intensity or myeloablative HCT from HLA-matched related or unrelated donors at their center tp 1997–2003 were selected. The patients were divided into a training set (two-thirds to develop scoring weights), and one-third of the patients were assigned to the validation set. Specific weight was assigned to comorbidities (**Table 1**) derived from Cox proportional hazards to predict the 2-year NRM in allo-HCT patients as the primary outcome. Adjusted hazard ratios for 2-year NRM were calculated for age and comorbid conditions while controlling for the intensity of conditioning regimen and disease risk. Comorbidities with adjusted hazard ratio of 1.2 or less were excluded, comorbidities with adjusted HR of 1.3–2.0 were assigned a weight of 1, comorbidities with adjusted HR of 2.1–3.0 or assigned a weight of 2 and comorbidities with adjusted HR of 3.1 or more were assigned a weight of 3. The final HCT-CI score was for some of these individual weights, and patients were stratified into low- (score 0), intermediate- (score 1–2), and high-risk (score ≥3).

**Table 1:** Definitions of comorbidities used for calculation of HCT CI.

Comorbidity	Definition of Co morbidity	HCT-CI-weighted scores
***Arrythmia***	Atrial fibrillation, flutter, sick sinus syndrome or ventricular arrhythmias	1
***Cardiac***	Coronary artery disease, congestive heart failure, myocardial infarction, ejection fraction ≤ 50%	1
***Inflammatory bowel disease***	Crohn’s disease or ulcerative colitis	1
***Diabetes***	requiring treatment with insulin or oral hypoglycemics	1
***Cerebrovascular disease***	Transient ischemic attack or cerebrovascular accident	1
***Psychiatric disturbance***	Depression or anxiety requiring psychiatric consultation or treatment	1
***Hepatic (mild)***	Chronic hepatitis with bilirubin up to 1.5 times upper limit of normal; AST/ALT up to 2.5 times upper limit of normal	1
***Obesity***	BMI ≥35	1
***Rheumatologic***	SLE, rheumatoid arthritis, polymyositis, mixed connective tissue disorder or polymyalgia rheumatica	2
***Peptic Ulcer***	Requiring treatment	2
***Renal***	Serum creatinine greater than 2 mg/dL, on dialysis or prior renal transplantation	2
***Pulmonary (moderate)***	DLCO and/or FEV1 66%–80%, dyspnea on slight activity	2
***Prior solid tumor***	Treated at any time in the patient’s history excluding nonmelanoma skin cancer	3
***Heart Valve***	Except mitral valve prolapse	3
***Pulmonary severe***	DLCO and/or FEV1 <65%, dyspnea at rest or requiring Oxygen	3
***Hepatic (moderate to severe)***	Liver cirrhosis, bilirubin greater than 1.5 ULN or AST/ALT greater than 2.5 ULN	3

The predictive value of HCT CI scores was confirmed in an independent cohort of patients (validation set), and the tool correctly predicted 2-year NRM and overall survival in patients (**Table 2**); moreover, its predictive accuracy was better than that of CCI. The major advancement with this new HCT-CI scoring system was refinement in the definition of comorbidities definition and introduction of objective laboratory and functional testing criteria that allow accurate assessment of comorbidities and replicability across independent observers.

**Table 2:** Hazard ratio for 2-year NRM and OS based on HCT-CI score.

Score	Number of patients	HR for 2-yr NRM	2-yr NRM	HR 2-yr OS	2-year OS
***0***	38	1.0	14%	1.0	71%
***1–2***	34	1.42 (0.8–2.7)	21%	1.31 (0.8–2.0)	60%
***≥3***	28	3.54 (2.0–6.3)	41%	2.69 (1.8–4.1)	34%

The HCT-CI score was subsequently validated at two large transplant centers in acute myeloid leukemia patients undergoing allogeneic transplantation in the first complete remission. On multivariate analysis, a high HCI-CI score was associated with the highest hazard ratios impacting NRM and overall survival among cohorts across both centers. This tool is now routinely used to assess comorbidities in patients before allogeneic stem cell transplantation. Online tools are now available for easy calculation of HCT-CI: http://www.hctci.org/.

**Figure 3:**
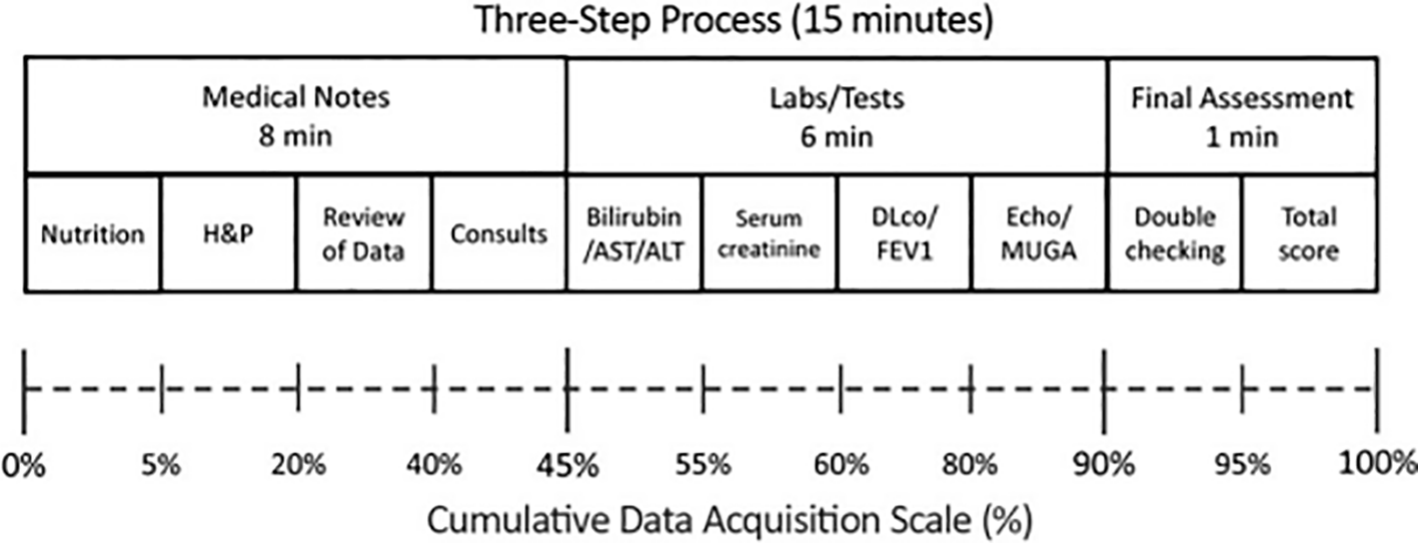
Recommended schema for calculation of HCT-CI.

To standardize patient assessment and data acquisition for calculation of HCT-CI, developers of this tool recommend a systematic three-step process that takes approximately 15 min (25) (**Figure 3**). On the Landmark assessment day (typically day −10), the first 8 min were used for evaluation of prior medical notes, evaluation of nutritional status, history and physical examination, review of laboratory data, and input from consultants. The next 6 min were spent on evaluating labs and tests including hepatic function, serum creatinine, pulmonary, and cardiac functions, and the final 1 min was spent calculating the total score (**Figure 1**). Guidelines for the assessment of organ function and assigning scores were provided by the developers of this tool. With the widespread use of HCT CI for the assessment of NRM prior to transplantation, HCT-CI has now been validated in non-myeloablative conditioning regimens (26), age index has been added (27), and validation has been performed at other transplant centers worldwide (28).

### Disease risk index

The disease risk index (DRI) for patients undergoing allo-HCT was first developed by investigators at the Dana-Farber Cancer Institute to account for variations in transplant outcomes based on disease-related variables, such as remission status and prognostic factors for hematologic malignancies (e.g., cytogenetic risk for AML) preceding transplantation (29, 30). Differences in disease risk are critical for correctly interpreting impact the impact of conditioning regimens or interventions in patients undergoing allo-HCT in both retrospective and prospective studies. Such a tool would also help stratify patients into risk groups and allow personalized of therapy in patients undergoing allo-HCT, e.g., recommendations for intensified condition regimens for patients with high relapse risk and de-escalation for patients with lower relapse risk. To develop this tool, a training set of 1,539 consecutive adult patients who underwent their first allogeneic stem cell transplantation using either reduced intensity (RIC) or myeloablative (MAC) conditioning between 2000 and 2009 were selected (31). The validation was performed using an external cohort of 672 patients from another large institution. For all patients enrolled in the study, pre-transplant information and subsequent transplant outcomes were collected from the database and electronic medical records were reviewed. Cytogenetics data were collected for patients with acute leukemia/MDS and CLL, and comorbidities were assessed using the HCT-CI score, which was available for a subset of patients transplanted between 2005 and 2009. Baseline characteristics of patients recruited in the training set were as follows: 53% of the patients underwent MAC, AML was the most common disease for which transplant was performed and 30% of the patients were in first remission at the time of transplant. Forty percent of the patients underwent allo-HCT from HLA-matched related donors (MRD), 45% of the patients underwent transplantation from matched unrelated donors (MUD), and 15% received mismatched unrelated donor transplantation. Most of the patients received peripheral blood stem cell grafts. The DRI to predict 4-year OS was calculated using Cox proportional hazard models, which included the following variables: age, sex of the donor and recipient, donor type, HLA match, graft source, CMV serostatus of donor and recipient, GVHD prophylaxis, therapy-related AML, FLT3 ITD status for AML, year of transplantation, and whether the transplant was performed on a clinical trial. Using this model, patients were stratified into four groups based on a 4-year overall survival of 64% (lowest-risk group; 15% of patients), 46% (intermediate-risk group 55% of patients), 26% (high-risk group, 27% of patients), and 6% (very-high-risk group 3% of all patients). In multivariable model, compared with intermediate risk group, the hazard rate for mortality associated low risk disease/status was 0.6, high risk was 1.8, and very high risk was 3.1 (all statistically significant). The DRI also stratified patients by 4-year PFS, ranging from 56% in the low-dose group to 6% in the very high-risk group (p <0.001). The strength of DRI is that it incorporates variables shown to be prognostic in allogeneic transplant outcomes: histologic subtype for lymphoma and cytogenetics for AML and MDS and remission status prior to transplantation for both.

### Risk stratification based on disease type

1. ***Low risk disease***: AML with favorable cytogenetics, CLL, CML, Hodgkin’s lymphoma, and non-Hodgkin lymphoma

2. ***Intermediate risk disease:*** ALL, AML or MDS with intermediate cytogenetics, myeloproliferative neoplasms, and multiple myeloma

3. ***High-risk disease:*** AML or MDS with adverse cytogenetics, and extranodal T-cell lymphomas.

### Risk stratification based on stage

**1. Low risk by stage**: CR 1, CR greater than 1(for MAC), PR 1, untreated disease, CML chronic phase.

**2. High risk by stage**: CR greater than 1(RIC), PR greater than 1, induction failure or active relapse, CML with accelerated or blast phase.

In this study, investigators found that HCT-CI was also independent prognostic factor for overall survival (along with DRI), suggesting that both should be used for the determination of clinical outcomes in patients undergoing a transplant consultation. This finding is consistent with other studies that have shown that patients with high-risk diseases have greater NRM (26, 32). Incidentally, patients with the highest DRI had higher HCT-CI scores in this study, most likely due to an increase in comorbidities associated with multiple attempts at remission induction with chemotherapy combinations.

The DRI was subsequently validated in a larger study of 13,000 patients who underwent allo-HCT between 2008 and 2010 and whose outcome data were reported to the CIBMTR. The DRI stratified patients into four groups with 2-year overall survival ranging from 64% to 24%. DRI was the strongest prognostic indicator for 2-year overall survival irrespective of age, conditioning regimen, graft source, or donor type (33). The DRI is now widely used for assessment of OS outcomes in allo-HCT patients undergoing HCT.A commonly used tool is an online calculator provided by CIBMTR that assigns DRI based on disease type, remission status, and cytogenetic risk.

https://cibmtr.org/CIBMTR/Resources/Research-Tools-Calculators/Disease-Risk-Index-DRI-Assignment-Tool

Despite its widespread adoption, further refinement based on additional information regarding risk stratification provided by molecular mutations in AML (34) and minimal residual disease status in pre-HCT evaluations (35, 36). Currently, these important disease-related factors and age are not captured in the DRI.

The European Group for Blood and Marrow Transplantation (EBMT) also developed a risk-scoring model for patients undergoing allo-HCT initially for CML, which was a common indication for allo-HCT when the score was first developed. Using patient- and disease-related features such as stage of CML, age/sex of donor/recipient and histocompatibility match, and time from diagnosis to allo-HCT, a scoring system was developed that accurately predicted clinical outcomes such as OS/LFS/RFS and NRM in CML patients (37). This score was subsequently validated for additional hematological malignancies in a large subset of allo-HCT patients (N = 56,505) with a median age of 33 years who underwent allo-HCT between 1980 and 2005. Using a seven-point scoring system (0–7), the EBMT score correctly predicted a 5-year OS of 71% for patients with a score of 0, and 24% for patients with a score of 6 or 7. All risk factors impacted survival and NRM, with increasing recipient age and MUD having a greater impact on aplastic anemia, time to allo-HCT impacted outcomes in NHL, and sex mismatch impacted NRM across all disease types (38). The decision to use risk scores at a center depends on institutional practices and familiarity with the scoring systems. All tools described above are validated in large sample sizes and can be used to help with HCT-related discussions with patients to ensure that they are aware of the risk benefit of allo-HCT. These scoring systems can also guide the choice of conditioning regimens, e.g., in patients with multiple comorbidities and high HCT-CI, RIC, or nom myeloablative regimens may be preferred compared to situations where patients have a high risk of relapse (39), where more intensive regimens may be chosen to improve RFS/OS (40).

### Risk stratification based on somatic mutations

Papaemmanuil et al. enrolled 1,540 patients from three prospective trials of AML patients between the ages of 18 to 65 years who received intensive therapy with anthracycline and cytarabine (41). Their genetic and cytogenetic profiles was obtained by sequencing 111 genes to identify driver mutations. Driver mutations were defined as recurrent fusion gene, aneuploidy, and leukemia gene mutations. They identified 5,234 driver mutations in 76 genes; one driver mutation was detected in 96% of the patients tested, and two or more driver mutations were detected in 86% of the samples. Based on acquired somatic mutations, patients with AML were genetically classified into 11 distinct categories. AML with NPM1 mutations is most seen in 27% of patients and is frequently co-mutated *with DNMT3A, FLT3-ITD, TET2*, and *PTPN11*. AML with mutated chromatin, RNA splicing genes, or both were detected in 15% of AML patients and frequently co-mutated genes were *RUNX1, SRSF2, MLL, DNMT3A, ASXL1, STAG2, NRAS, TET2*, and *FLT3-ITD*. AML with *TP53* mutations was observed in 13% of the patients and was associated with a complex karyotype, monosomy 5, monosomy 7, 17p deletion, trisomy 8, and 12p deletion. Other subgroups were as follows: AML with inversion 16 or t(16;16) (*CBFB-MYH11*), AML with allelic CEBPA mutations, AML with t(15:17) *PML-RARA*, AML with t(8;21) *RUNX1-RUNX1T1*, AML with *MLL* fusion genes, AML with inversion 3 *(GATA2*) or t(3;3) *MECOM*, AML with *IDH-2* mutations, and AML with t(6;9) *DEK-NUP214.* This genetic calcification in females has prognostic clinical applications as patients with driver mutations in the chromatin-spliceosome group were older, presented with lower WBC counts and blast counts, and had lower response rates of induction chemotherapy, high relapse rate, and poor long-term clinical outcomes similar to patients in adverse risk groups. The most recent European Leukemia Net AML guidelines published in 2022 incorporated somatic mutations to describe AML/MDS with recurrent genetic abnormalities, taking precedence over other cytogenetically defined categories (34). In patients with AML defining recurrent genetic abnormalities, a blast count of 10% is required and sufficient for AML diagnosis, except in cases of BCR-ABL1 AML where a 20% myeloid blast threshold is still needed to avoid overlap with the accelerated phase of CML. Patients are now classified into favorable, intermediate, or adverse risk groups based on a combination of somatic mutations and cytogenetic abnormalities (**Table 3**). In this recent classification, the categorization of AML with myelodysplasia-related changes was removed as this has been supplanted by recurrent genetic abnormalities associated with prior therapy (del 5q/del7q or MLL gene rearrangements). Prior treatment exposures are now used as diagnostic qualifiers. A separate category of myeloid neoplasm associated with germline predisposition has also been made in the most recent guidelines, given the increasing recognition of germline mutations (*DDX41, TP53, ETV6, GATA2, NF1, PTPN11*, etc.) in causing hematologic malignancies.

**Table 3:** Risk stratification of AML based on cytogenetic and molecular features.

Risk Category	Genetic Abnormality
**Favorable**	➢ t (8;21) (q22; q22.1)/*RUNX1-RUNX1T1* ➢ inv (16) (p13.1q22) or t (16;16) (p13.1; q22)/*CBFB-MYH11* ➢ *NPM1*m without *FLT3-ITD* ➢ bZIP mutated *CEBPA*
**Intermediate**	➢ *NPM1*m/wt with *FLT3-ITD* ➢ t (9;11) (p21.3: q23.3)/*MLLT3:KMT2Ar*
**Adverse**	➢ t (6;9) (p23.3; q34.1/*DEK-NUP214* ➢ t(v;1q23.3)/*KMT2Ar* ➢ t (9;22(9q34.1; q11.2)/*BCR-ABL1* ➢ t (8;16) (p11.2; p13.2)/*KAT6A-CREBBP* ➢ inv (3) (q21.3; q26.2) or t (3;3) (q21.3; q26.2) *GATA2/MECOM* ➢ t (3q26.2v)/*MECOM* ➢ *-5 or del(5q); -7; -17/abn(17p)* ➢ *Complex karyotype or monosomy karyotype* ➢ *Mutated ASXL1, BCOR, EZH2, RUNX1, SF3B1, SRSF2,STAG2,U2AF1,ZRSR2* ➢ *Mutated TP53*

Patients with newly diagnosed AML should undergo extensive cytogenetic, molecular, and immunophenotyping for accurate risk assessment at diagnosis at a center with expertise in performing these sophisticated tests. This initial assessment is crucial for accurate risk assessment, initiation of induction therapy based on the presence of somatic mutations, e.g., FLT3-ITD, IDH mutations or KMT2A rearranged AML or CBF AML, and in making decisions regarding post-induction consolidation strategies. Studies have shown no adverse effects of waiting for molecular testing to complete therapy (42).

### MRD (measurable residual disease) for risk stratification of AML

MRD refers to residual disease detectable in patients with hematologic malignancies (AML/ALL) measured by specialized techniques; otherwise, it is in morphologic remission, as noted by conventional techniques. Multiple studies have shown a poor prognostic impact of pre HCT MRD positivity and allo-HCT outcomes (43). Acute myeloid leukemia MRD is evaluated using multiparameter flow cytometry and PCR-based testing for specific mutations (*NPM1*) or fusions (CBFB*-MYH11*, etc.). An emerging area of research is next-generation sequencing (NGS)-based analysis for MRD detection. The ELN has made specific recommendations regarding MPFC-based analysis for the accurate production of MRD (44). A diagnostic bone marrow sample is recommended for the evaluation of core markers including CD33, CD34, CD117, CD45, CD13, CD56, HLA-DR, and CD7. When assessing the immunophenotype of remission marrow, particular attention needs to be paid to cells that are different from normal (DfN) or have an immunophenotype similar to that of diagnostic bone marrow biopsy. Often, the combination of both DfN and leukemia-associated immunophenotype (LAIP) approaches is needed, and if there is uncertainty regarding residual disease due to overlap with recovery marrow, this needs to be reported as well. To obtain accurate results from bone marrow aspirate for MRD analysis, 3 mL from the first pull of the bone marrow biopsy is recommended to prevent hemodilution from subsequent aspirate specimens. Cellular viability is critical for correct interpretation, and samples should be shipped to the reference laboratory at the earliest, as delays of greater than 3 days affect diagnostic accuracy. To obtain accurate results, >500,000 CD45 cells and greater than 100 viable cells must be assessed in the blast gate for MRD reporting. The diagnostic accuracy of the MPFC based on MRD analysis ranged from 0.1% to 0.01%. For molecular MRD analysis, PCR- and NGS-based assays were employed. For optimal results, 5 mL of bone marrow aspirate from the initial pull was sent for analysis. For NGS-based MRD testing, germline mutations (*DDX41, GATA2, RUNX1, TP53*, etc.) and CHIP mutations (DNMT*3A, TET2* and *ASXL1*) should be excluded. Similarly, mutations in signaling pathways (*FLT3 ITD/TKD, KIT RAS*) should be excluded, as they may represent subtotal populations of cells and have low negative predictive value. Currently, there is no validated NGS panel for MRD testing; however, efforts are ongoing to standardize these tests in a prospective trial (MEASURE). Currently, PCR-based approaches for MRD testing and AML patients are limited to approximately 40% to 60% of patients harboring targetable mutations, including *NPM1, RUNX1-RUNX1T1, CBFB-MYH11, PML-RARA, KMT2A-MLLT3, DEK-NUP214, BCR-ABL*, and *WT1*. Recent data suggest that PCR-based approaches for certain mutations (NPM-1 and FLT3-ITD) have prognostic value in relapse prediction, indicating the importance of NGS-based testing as an MRD tool (45). For these mutations, either bone marrow or peripheral blood may be used for MRD analysis given the high sensitivity of these assays in the range of 10^−4 to −6^. Patients with persistent MRD positivity after induction chemotherapy either by flow cytometry, molecular or a combination of these techniques, have a high risk of relapse after allogeneic stem cell transplantation, and strategies for MRD clearance include intensification of conditioning regimen (36), addition of novel drugs to eliminate MRD, or using posttransplant maintenance strategies (46, 47). Studies have also suggested that MRD-positive disease graft selection may be important, as cord blood HCT (48) and haploidentical HCTs (49) may be preferred donor choices in these settings. These differences between HCT outcomes based on donor type may be related to more effective leukemia stem cell (LSC) clearance by immune cells due to differences in cytokine profiles, resulting in immune reconstitution and superior GVL with haploidentical and cord blood HCT (50, 51).

In conclusion, the assessment of variables that have been validated to impact HCT outcomes is crucial to help both patients and physicians better understand the relative risk benefit of HCT. This will allow treating physicians to make educated decisions regarding escalation or de-escalation of conditioning regimens based on patient-related (age, comorbidities, HCT-CI score) and disease-related factors (DRI,EBMT score, MRD status before HCT, and somatic mutations at diagnosis). Accurate risk assessment may also influence the choice of GVHD prophylaxis and maintenance strategies to improve the GRFS outcomes. All available tools, such as DRI, HCT-CI, and EBMT scores, are validated in large patient cohorts for all common hematologic malignancies, and the decision to use one versus the other often depends on physician choice and institutional practice.

## Donor selection

Some of the key factors that impact transplant outcomes are the underlying hematological conditions, recipient comorbidities, and suitables donor. Selection of an appropriate donor is crucial for successful transplantation.

Patients’ human leukocyte antigen (HLA) typing should be performed once a diagnosis of a hematological condition is made for which stem cell transplantation is indicated. Family history should be obtained, and if patients have full siblings (same parents), they should be typed, as this is the quickest way to identify HLA-matched donors.

### Recipient–donor HLA matching

HLA is a major histocompatibility complex (MHC) system located on the short arm of chromosome 6 that contains the most polymorphic genetic region of the entire human genome (52). Each parent inherits a group of HLA genes called a haplotype. HLA matching is a single-paramount criterion for selecting suitable donors. Pertinent genes for allogeneic transplantation (allo-HCT) in class I (HLA-A, HLA-B, and HLA-C) and class II (HLA-DR, HLA-DQ, and HLA-DP). Several large studies have shown that a single mismatch at HLA-A, B, C, and DR is associated with higher mortality compared with 8/8 matched donors (53).

### Matched related donors (MRD)

Each individual had two HLA alleles inherited by each parent. Therefore, the probability of full siblings sharing the same haplotype was approximately 25%. HLA-matched sibling donors are the best donors for patients undergoing allo-HCT. Several studies on a variety of diseases have shown that MRD is associated with the best survival. However, the HLA 8/8 allele-matched URD had similar TRM and all-cause mortality compared to MRD. However, MRD is associated with a lower risk of acute and chronic GVHD, which is a serious and common complication of allo-HCT (54–56).

### Matched unrelated donors (MUD)

In patients with no full siblings, concurrent MUD is initiated once allele-level HLA typing is available. Although MRD is considered the best donor, it is only available in less than one-third of the patients. Thus, the MUD is an acceptable alternative. Currently, MUD worldwide registries have more than 40 million volunteer donors (https://wmda.info/). Recent advances include precise HLA-typing, supportive care, and immunosuppressive strategies. This can be shown by the NMDP data of one-year improvement in survival (**Table 4**).

**Table 4:** Overall survival outcomes post MUD transplant based on era.

Improved Survival with Unrelated Transplantation	
YEAR OF HCT	ONE-YEAR SURVIVAL
2013-2015	66%
2010-2012	61%
2007-2009	59%
2004-2006	50%
20001-2003	45%
1998-2000	43%

Transplant outcomes using MUD are now comparable to those using MRD in several patient populations (57). With an increase in size and diverse ethnic background inclusions in various worldwide stem cell donor registries, most patients without a MRD can proceed to transplantation using MUD. However, this probability differs for various ethnic backgrounds.

### Alternative donors

When an MRD or MUD is not available, an alternative donor search includes the following:

#### Mismatched unrelated donors (mMUD)

Less than 8/8 HLA A, B, C, and DR is considered mMUD. This may be due to antigen and allele levels. Several large and registry retrospective studies have shown worse outcomes with a greater number of mismatches with calcineurin-based graft-versus-host disease (GVHD) prophylaxis. This has led to improvements in GVHD prophylaxis and the use of post-transplant cyclophosphamide (PTCy) which is now commonly used. Other strategies include CD34+ cell-selective T-cell depletion as well as other graft manipulations such as CD34+ selection and add back memory CD45RA+ T, α/β T-cell depletion (58).

#### HLA-haploidentical donors (HHD)

Recent advances, including T-cell depletion and PTCy-based regimens, have allowed the use of partially matched related donors as viable options for allo-HCT. HHD are related donors (first-degree relatives such as parents, sibling, children, or extended families including uncles, aunts, cousins, nephews, and nieces) that share one HLA haplotype and mismatch at other HLA haplotypes by a variable number of HLA genes. Several retrospective studies have shown comparable outcomes between haploidentical transplants and MUD transplants (59, 60).

#### Umbilical cord blood transplant UCB

**Figure 4:**
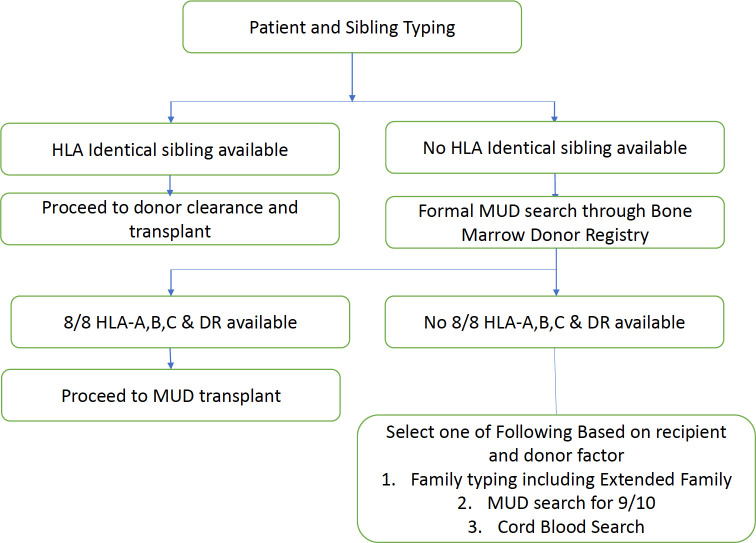
Donor selection algorithm based on HLA typing.

UCB is another alternative if there is no available MRD or MUD. It offers several advantages over the MUD. There is more room for HLA mismatch, so 1–2 mismatches, relatively quicker time to graft identification and acquisition, and no risk to the donor. However, some of the limitations are limited cell dose, no option for second collection, and donor lymphocyte infusion (DLI). In terms of transplant, a higher infectious risk is due to slow immune reconstitution, count recovery, and graft failure. The number of UCB transplants has recently declined owing to the increased use of HHD (61). Our approach to donor selection is illustrated in **Figure 4**.

### Other factors to look for donor selection

In addition to HLA typing, the following factors are important when selecting an appropriate donor. A detailed discussion of all the factors is beyond the scope of this review; however, the important features are discussed below. The reader is referred to previously published excellent reviews for donor selection (62, 63).

#### Anti-HLA antibodies

All patients undergoing donor search should undergo HLA-antibody screening to detect donor-specific anti-HLA antibodies (DSA). DSA is associated with an increased risk of graft failure and decreased survival (64). Therefore, if a patient has DSAs, every effort should be made to identify an unrelated donor or even a UCB graft to which the patient has not been sensitized. This is particularly more important in HHD, especially in the child donor-to-mother recipient setting, as mothers are likely to be allosensitized and form antibodies against mismatch donor HLA antigens from pregnancy (64). However, if no other donor options are available and DSA has a low concentration, desensitization should be attempted to lower DSA and improve engraftment after allo-HCT (65).

#### Donor age

Large retrospective studies have shown an improvement in survival after MUD in younger donors compared to older donors. There was a 5.5% increase in the HR for overall mortality for revery 10 years increasement in donor age. Similarly, data from large retrospective studies have shown that an increase in donor age by a decade was associated with a decrease in OS, PFS, and a higher risk of acute GVHD but not chronic GVHD (66). Recent data show that younger patients with HHD have better outcomes compared to ≥35 MRDs and MUDs due to a lower risk of chronic GVHD and relapse-free survival (67).

#### CMV status

CMV seropositivity is determined in both the recipient and the donor. Matching the patient/donor CMV status affects transplant outcomes. Some studies have shown that the risk of CMV reactivation is more common after allo-HCT in CMV mismatches between the donor and recipient (68, 69). However, this may not be as significant as FDA approval and use of letermovir for CMV prophylaxis in CMV seropositive recipients (70).

#### ABO and Rh status

Compared with solid organs, allo-HCT can be safely performed despite ABO/Rh incompatibility. However, this incompatibility may result in complications, including hemolysis, slow red cell engraftment, GVHD, and PRCA (56, 71). Therefore, if multiple donors are available, ABO compatibility is preferable to reduce the risk of the above-mentioned complications.

#### Killer immunoglobulie-like receptor (KIR) status

Natural killer (NK) cells are part of the innate immune system and play a key role in the early immune response against infections and tumors. In allo-HCT settings, donor NK cells can exert a GVL effect when they express inhibitory KIR that does not engage with recipient cell MHC class I molecules (62). NK cell alloreactivity was assessed by killer KIR genotyping. KIR genes can be divided into two broad haplotypes, KIR A and KIR B. KIR A contains one activating receptor, whereas KIR B contains two or more activating receptors. Individuals can be divided into two different KIR haplotypes: KIR A/A and KIR B/X (72, 73). The donor B haplotype confers better protection against relapse and improved DFS than the donor A haplotype. In addition, the clinical benefits increase with the number of B-specific gene motifs.

## Mobilization and collection of peripheral blood stem cells

The collection of a sufficient number of hematopoietic stem cells (HSC) is crucial for ensuring timely and sustained engraftment following hematopoietic cell transplantation (HCT). The generally accepted minimal HSC dose is 2 × 10^6^ CD34^+^ cells/kg recipient body weight (74, 75). The “ideal” target dose was less well defined. Studies have reported an association between higher doses of infused CD34+ cells with faster hematopoietic recovery and lower resource utilization (76, 77), but also with an increased risk of severe graft-versus-host disease following allogeneic HCT (78). In general, a target collection dose of 3–5 × 10^6^ CD34^+^ cells/kg is recommended for autologous HCT, and 5-8 × 10^6^ CD34^+^ cells/kg is recommended for matched donor allogeneic HCT (79). However, it is also reasonable to accept a lower collection yield, e.g., 3.0 × 10^6^ CD34^+^ cells/kg for allogeneic HCT, instead of prolonging the mobilization and extending the collection over multiple days for the donor to reach a higher CD34+ cell dose (74).

Most HSCs reside in the bone marrow (BM). However, BM harvesting of HSCs is accompanied by procedural risks and lower CD34+ cell yields (80). Alternatively (and more commonly), HSCs are “mobilized” from the BM into the peripheral blood to allow the collection of peripheral blood stem cells (PBSCs) *via* apheresis. In 1986, it was shown that the administration of chemotherapy resulted in a temporary increase in the number of stem cells in peripheral blood during hematopoietic recovery (81). Subsequently, it was shown that such mobilization in autologous donors can be further enhanced with the use of myeloid growth factors such as granulocyte colony-stimulating factor (G-CSF, filgrastim, pegfilgrastim), or granulocyte–macrophage colony stimulating factor (GM-CSF, sargramostim), either alone or following chemotherapy (82). G-CSF is also the most potent, and commonly used commercially available growth factor to mobilize allogeneic PBSC donors. G-CSF stimulates neutrophil production and maturation and facilitates the release HSCs into the peripheral blood by inducing the release various proteases in the marrow, thereby disrupting the adhesion of CD34+ cells to the BM stroma (83). G-CSF is typically administered subcutaneously to the donor at a dose of 10 µg/kg/day for 4–5 days prior to apheresis collection. The dose may be rounded up or down to facilitate ease of administration and minimize waste, as G-CSF is typically supplied in 300- or 480-µg vials.

However, conventional mobilization regimens are associated with mobilization failures or inability to collect enough PBSCs in 5%–40% of autologous HCT candidates (84) and up to 5% of healthy allogeneic donors (85). Risk factors include advanced age, radiation of the marrow, BM involvement by the underlying disease, and prior treatment with marrow-toxic agents such as lenalidomide, purine analogs, and alkylating agents (85). Systemic factors such as stress, cortisol level, trauma, infection, and inflammation, interactions with the coagulation and complement cascades, and signals from the central and sympathetic autonomic nervous systems are also thought to affect mobilization (86).

Plerixafor (AMD 3100, or Mozobil, Sanofi, Cambridge, MA) is a newer but valuable addition to the arsenal of mobilization agents. Mechanistically, plerixafor is synergistic with G-CSF and enhances the release of HSCs into peripheral blood by disrupting the adhesion between CXCR4 expressed on CD34+ cells and its ligand CXCL12 (also known as stromal-derived factor-1) expressed by BM stromal cells (87). Initially approved by the FDA in 2008, following two Phase III prospective randomized trials in NHL and MM patients (88, 89), extensive literature has since emerged documenting its efficacy in patients with a growing list of additional diagnoses, including Hodgkin’s lymphoma, germ cell tumors, and various non-hematologic diseases (90–93), as well as its successful use in allogeneic donors with poor PBSC mobilization (94).

For optimal efficacy, plerixafor is administered subcutaneously in the evening, preferably 10–14 h prior to each apheresis collection session occurring the following morning (95), at a dose of 0.24 mg/kg body weight, or 0.16 mg/kg for patients with creatinine clearance of 50 mL/min or less. Because plerixafor is supplied in 24 mg single-use vials, for allogeneic donors weighing above 100 kg, the dose can be capped at 24 mg, rather than using an additional vial. This dose-capping strategy has been shown to confer significant cost-savings and achieved comparable collection outcomes as administering uncapped doses in patients weighing >100 kg and collecting autologous PBSCs (96).

Plerixafor has been demonstrated to be effective and safe in healthy donors when administered as the sole mobilization in a single dose shortly before collection, favorable collection outcomes have been observed in multiple studies (97–100). However, at over $8,000 per single-use vial at most hospitals, based on contracted wholesale prices (96), the main factor limiting the routine use of plerixafor in donors is the cost. In general, the use of plerixafor in allogeneic stem cell donors is more practical as an add-on salvage agent when there is a need to quickly collect an adequate amount of stem cells in a donor with suboptimal mobilization, whether due to the medical urgency of a conditioned recipient waiting for transplant, or limited donor availability or risk tolerance for further apheresis or bone marrow collection. The added cost should be balanced against the gains in greater mobilization success rate, higher CD34+ cell yields, and savings, as well as donor comfort and convenience associated with fewer collection days, or avoidance of a BM harvest compared to using conventional regimens. The urgent use of plerixafor is also justified when the collection yield is too low to proceed with the planned transplant and the recipient has already been conditioned. Collection facilities should develop strategies and algorithms for plerixafor use to optimize the collection success rate and cost-effectiveness.

At our center, over an 8-year period, 4.1% or 41 of the 1,008 allogeneic donors received one dose of plerixafor in addition to G-CCSF due to poor collection yield after one day of collection, generally <60% of the desired collection target, or <2.0–2.5 × 10^6^ CD34^+^ cells/kg of recipient body weight. After starting plerixafor, there was a 0.75- to 7.74-fold increase in the CD34+ yield from the previous day. The median 2.94-fold increase with plerixafor was similar to that reported in previous studies evaluating the use of plerixafor in allogeneic donors (101, 102). Among donors who collected <2.0  × 10^6^ CD34+ cells/kg recipient weight on day one, none of those who received G-CSF-only mobilization but no plerixafor achieved the goal of ≥4.0 × 10^6^ CD34+ cells/kg recipient weight over 2 days, but 59.2% of donors who received rescue plerixafor did. The selection of plerixafor has allowed the vast majority of donors to collect sufficient CD34+ cells to proceed with allogeneic HCT for the recipient at our center with an adequate cell dose (94).

In conclusion, the primary goal of PBSC mobilization and collection is to obtain sufficient HSCs to allow for successful and durable engraftment following HCT. This can be accomplished with the use of conventional regimens of myeloid growth factors such as G-CSF in allogeneic donors, and the judicious use of adjunct agents such as plerixafor when mobilization is suboptimal. Assessment of the risk factors for inadequate mobilization and close monitoring of the mobilization and collection progress in donors can significantly improve the collection success rate while maintaining cost-effectiveness. The criteria for using plerixafor in allogeneic donors should be developed to allow individualization in specific cases. Centers should develop their own specific criteria and triggers for plerixafor use based on evidence in the literature and their own clinical data, which should also be continuously assessed and validated using institutional data and donor collection outcomes.

## Conclusion

Recent CIBMTR data suggest that trends in allo-HCT are changing, which may change the practice in the coming years. Increasingly, older patients are being transplanted using PBSC grafts with unrelated donors. These practice changes indicate that the incidence of acute and chronic GVHD will continue to rise, increasing the clinical burden of HCT-related complications. Fortunately, new drugs, such as PTCy, use of JAK inhibitors and *ex vivo* graft manipulation strategies, have been approved for the treatment of established cGVHD and novel strategies for prevention. The success of allo-HCT depends on appropriate donor selection, and we discuss our strategy for the selection of sibling, unrelated, and alternative donors. Nationwide, the utilization of cord blood HCTs is declining and haplo-HCTs are increasing. Donor age is now recognized as an important variable determining the success of HCT in both matched donors and haploidentical HCTs, and this is now one of the determining factors in choosing a donor for allo-HCT. Finally, we discuss strategies for HPC collection in an allogeneic setting, focusing on the role of plerixafor in patients who mobilize poorly using G-CSF alone.

## Author contributions

AS conceived, wrote and reviewed the manuscript. SY and HA contributed to writing the manuscript. All authors contributed to the article and approved the submitted version.
